# Dengue Virus Tropism in Humanized Mice Recapitulates Human Dengue Fever

**DOI:** 10.1371/journal.pone.0020762

**Published:** 2011-06-10

**Authors:** Javier Mota, Rebeca Rico-Hesse

**Affiliations:** Department of Virology and Immunology, Southwest Foundation for Biomedical Research, San Antonio, Texas, United States of America; Centers for Disease Control and Prevention, United States of America

## Abstract

Animal models of dengue virus disease have been very difficult to develop because of the virus' specificity for infection and replication in certain human cells. We developed a model of dengue fever in immunodeficient mice transplanted with human stem cells from umbilical cord blood. These mice show measurable signs of dengue disease as in humans (fever, viremia, erythema and thrombocytopenia), and after infection with the most virulent strain of dengue serotype 2, humanized mice showed infection in human cells in bone marrow, spleen and blood. Cytokines and chemokines were secreted by these human cells into the mouse bloodstream. We demonstrated that the pathology of dengue virus infection in these mice follows that reported in human patients, making this the first valid and relevant model for studying dengue fever pathogenesis in humans.

## Introduction

Dengue has become the most important mosquito-transmitted virus to human health since it is now endemic in over 100 countries and infects over 50 million persons per year. Dengue viruses, of which there are 4 antigenic types or serotypes, cause dengue fever (DF), a self-limiting, influenza-like illness with rash, or dengue hemorrhagic fever (DHF), a much more severe disease, with bleeding abnormalities that can lead to death. The large majority of human cases present with DF, some with minor bleeding abnormalities, and about 1% of patients, mainly children, go on to develop DHF and require hospitalization [1]. However, little is known about the mechanisms of dengue pathogenesis and there are no treatments nor prophylaxis for dengue infection; currently, the only method of disease control is mosquito abatement. Most of what is known about dengue disease progression comes from observational studies in human patients because there are no other hosts of this virus. Studies done in the early 20th century demonstrated that when a broad range of animals were inoculated with dengue virus, none of them became ill nor had high levels of virus in their blood (for a review see [2]). Thus, we have not had an animal model of disease in which to study the details of disease progression, and what we know to date is that human host and viral genetics are determinants of disease and that prior immunity to one serotype increases the probability of developing DHF. That is, antibodies or memory T cells to one virus may cause severe, hemorrhagic manifestations during a subsequent, heterologous virus infection, referred to as immune enhancement of disease [3], [4]. This phenomenon makes the study of dengue virus pathogenesis even more complex, given the fact that there are 4 dengue serotypes and a wide variety of genetic differences between strains within each serotype [5].

Immunodeficient mouse strains that have been transplanted with human stem cells (from umbilical cord blood or fetal cells) have been shown to develop functional human immune systems, including some level of adaptive immunity [6]. It has been reported that some of these mouse strains develop immunoglobulins specific for human immunodeficiency virus and dengue virus, albeit at low levels, after experimental infection [7], [8]. Only one humanized mouse model, receiving human fetal stem cells, has reportedly produced specific antibodies to dengue viruses after simultaneous infection with multiple strains of virus [9]. In contrast, we were the first to present results of dengue virus pathogenesis studies in the NOD/SCID mouse strain [10], and later, in the NOD-*scid IL2Rγ* mouse strain that has a higher degree of human lymphocyte development (median of 52% engrafment in blood) but after transplantation with human cord blood stem cells only [11]. We compared viruses from different genetic subgroups (genotypes) of dengue serotype 2 (DEN-2) and showed that there were significant differences in mouse clinical signs that correlated with infection by each of the 4 genotypes. Thus, we developed a measure of virulence in an animal model, a first for dengue, and these mice consistently present with signs of dengue fever as in humans. However, these mice do not develop specific antibodies to these viruses, and this is probably why they do not develop more severe disease, such as human-like DHF. In this report we present results of an in depth analysis of the pathology and tropism of dengue virus infection in the same strain of mice, using one strain of dengue virus serotype 2 (K0049, Southeast Asian genotype). A total of 27 humanized mice were infected and sacrificed during different time points after infection (9 independent experiments with n = 3 each), to define the dynamics of dengue virus replication and tropism in this mouse model of DF.

## Materials and Methods

### Ethics Statement

This study was carried out in strict accordance with the recommendations in the Guide for the Care and Use of Laboratory Animals of the National Institutes of Health. The protocol was approved by the Institutional Animal Care and Use Committee of the Southwest Foundation for Biomedical Research (Protocol Number: 1054 MU). All procedures were performed under isofluorane deep anesthesia, and all efforts were made to minimize suffering.

### Mice and transplantation

We established a colony of NOD.Cg-*Prkdc^scid^ IL2rg^tm1Wjl^*/SzJ (NSG mice) from breeding pairs obtained from The Jackson Laboratory (Maine), housed in a specific pathogen-free facility under sterile conditions. Animal manipulation and procedures were as described previously [11]. For transplantation, human cord bloods from anonymous donors were obtained from the South Texas Blood and Tissue Center (San Antonio, Texas) and peripheral blood mononuclear cells (PBMCs) were separated out by Ficoll-Hypaque density gradient followed by positive selection of CD34^+^ hematopoietic stem cells using a CD34^+^ Progenitor Cell Selection System (Invitrogen) according to the manufacturer's instructions. We used flow cytometry analysis (described below) to confirm purity, which usually ranged from 85 to 90%. In preparation for transplantation, newborn mice were transported in microisolation irradiator cages (Braintree Scientific) to the University of Texas Health Sciences Center facilities (San Antonio, Texas) and sub-lethally irradiated with 100 cGy using a Cesium source. Mice were then transplanted by intrahepatic inoculation of 3×10^5^ purified cord blood CD34^+^ cells. Mouse irradiation and transplantation procedures occurred 24–48 hrs after birth.

### Dengue virus preparation

Low passage dengue virus serotype 2, strain K0049 was used in this study. Viral stocks were prepared as described previously [11]. Briefly, confluent monolayers of the C6/36 (*Aedes albopictus*) grown in 75 cm^2^ flasks were infected with dengue virus at a multiplicity of about 10 genome equivalents (0.01 PFU) per cell and incubated 1 hour at 28°C with 5% CO_2_, after one wash, fresh medium was added and incubated for 7–9 days, until infection occurred in more than 90% of the cells as evidenced by indirect fluorescent antibody (IFA) assay, using mouse hyperimmune ascitic fluid of MAb 3H5, against DEN-2, specifically the E protein (CDC, Fort Collins, CO). Cell supernatants were harvested and stabilized by adding 30% (v/v) of a 2% gelatin solution (Sigma); 0.5–1.0 mL aliquots were prepared and stored at −70°C until use. Concentration in genome equivalents per mL (gEq/mL) was measured by real time quantitative RT-PCR and usually ranged from 1×10^9^ to 1×10^10^ gEq/mL. Only fresh, unthawed aliquots were used in all experiments.

### Flow cytometry

Purity of enriched CD34^+^ cells from cord blood was evaluated by flow cytometry, by staining cells with PE-conjugated anti-human CD34 (clone 563) antibody (BD-Biosciences), which is different from that used on beads for positive selection. To evaluate the presence of human cells in peripheral blood or mouse tissues, the following anti-human antibodies were used: CD45-APC, CD45-V500, CD3-Pac Blue, CD8-PerCP.Cy5.5, HLA-DR- PerCP (BD-Biosciences), CD16-Alexa Fluor 700, CD45-FITC (Invitrogen), CD20-FITC, CD14-PE (Beckman Coulter), and CD19-Alexa700 (Biolegend). To test engraftment levels 6 weeks after transplantation, 50 µl of blood was collected by the retro-orbital route using calibrated capillary micropipettes (Drummond); blood was deposited in heparinized collection tubes (Braintree Scientific) and stained with the antibodies listed above. Red blood cells were lysed with 1× Red Blood Cell Lysis Solution (Miltenyi), washed twice in PBS, fixed with 1.6% methanol-free formaldehyde and processed for flow cytometry analysis. To analyze human infected cells in different mouse tissues, blood, liver, lymph node, spleen and bone marrow (obtained by flushing the mouse femurs with RPMI supplemented with 10% of FBS) were obtained at different time points and single cell suspensions prepared by passing the cells through 100 µM cell strainers (BD-Biosciences). These cells then were counted using trypan blue, and one million used for surface staining with the antibodies listed above. For intracellular staining, cells were fixed and permeabilized with Cytofix/Cytoperm solution (BD-Biosciences) and stained for dengue antigen with MAb 2H2 (anti-prM gene) coupled to FITC [12]. Isotype-matched antibodies were used for appropriate surface and intracellular controls. Samples were acquired using a CyAn ADP analyzer and data were analyzed using Summit software (Beckman Coulter).

### Mouse infection and clinical sign monitoring

Adult mice (6–8 weeks) with engraftment levels of 17–89% were used to make experimental groups of 3 individuals each for infection experiments with DEN-2 virus. Viral stock was diluted with sterile PBS, to a concentration of 9 log_10_ genome equivalents (about 6 log_10_ PFU) in a final volume of 100 µl, and mice were inoculated intradermally on the back, while under isofluorane anesthesia. Mock-infected groups were inoculated with the same amount of C6/36 cell culture media prepared with 30% gelatin (v/v), as in the virus stock, and diluted in PBS. Mouse temperature and erythema were measured at the specified intervals (daily, initially, and on alternate days, later) and platelets were measured in infected and control mice at day 10 p.i. For temperature measurements we used a RET-3 rectal probe coupled to a BAT-12 Microprobe Thermometer (Physitemp Instruments), and erythema was measured using a DSMII ColorMeter (Cortex Technology, Denmark), as previously described [10], [11]. Viremia was measured in 10 µl of sera (from 25 µl of peripheral blood, obtained retro-orbitally) at the specified intervals, starting at day 1 p.i.

### Quantitative RT-PCR

RNA copies in viral stocks or in sera obtained from infected mice were measured as previously reported [11]. Briefly, the RNA template (5 µl of viral stock or 10 µl of serum) was amplified in duplicate or triplicate (sera or viral stock, respectively) using the RNA Ultrasense, One-step Quantitative RT-PCR System (Invitrogen), with the forward and reverse primers: d2C16A (5′-GCTGAAACGCGAGAGAAACC-3′), d2C46B (5′-CAGTTTTAITGGTCCTCGTCCCT-3′), and the VICd2C38B probe (FAM-5′-AGCATTCCAAGTGAGAATCTCTTTGTCAGCTGT-3′-TAMRA). This protocol amplifies a 94 bp fragment of a highly conserved region of the capsid gene of DEN-2 viruses and efficiently detects dengue virus in peripheral blood mononuclear cells from human clinical samples [13]. To estimate RNA copies, a standard curve was generated using 10-fold serial dilutions of *in vitro* transcribed RNA standards [14]. The sensitivity of the assay is 240 RNA copies per ml or 1.2 copies per reaction tube.

### Dengue ELISA

To determine the presence of human antibodies (total immunoglobulins) against DEN- 2 in the sera of humanized mice, we used an indirect ELISA as previously described [11]. Serum samples from infected and control mice were obtained as described above at different time points, serially diluted starting at 1∶20, and tested in duplicates. The cutoff to consider a sample positive was the average of the O.D. of negative controls plus one standard deviation.

### Cytokine Production

Human cytokines and chemokines were measured in the sera of infected and control humanized mice by means of multiplex analysis, with fluorescently-labeled microsphere beads in a Luminex platform. A panel of nine human cytokines and chemokines, TNF-α, IFN-γ, IL-2, sIL-2R, IL-6, IL-8, IL-10, MCP-1 and VEGF, were tested using the premixed Milliplex MPXHCYTO-60K kit (Millipore) according to the manufacturer's instructions. At different time points, serum samples from infected and control mice were obtained as described above, diluted 1∶1 and tested in duplicates using the Luminex 100 system (Luminex Co.), and the MasterPlex QT v4.0 software (MiraBio). Results were expressed in picograms per milliliter (pg/mL) and the average background of control samples was subtracted from results of infected mice.

### Histopathology

Liver, lymph node, spleen and femurs (for bone marrow staining) were collected at different time points and either embedded in optimal cutting temperature compound (OCT) and snap-frozen, or fixed in 10% neutral buffered formalin and embedded in paraffin. Frozen tissues were cut in 6 µM sections, fixed with 4% formaldehyde and permeabilized with 0.25% Triton X-100 in PBS. Sections were stained for human lymphocytes with an anti-human CD45-FITC (Invitrogen) diluted 1∶100 and for dengue virus using the anti-DEN-2 3H5 (anti-E protein) MAb (diluted 1∶200) as primary antibody, followed by Alexa Fluor-594 goat anti-mouse IgG (Invitrogen) as secondary antibody (diluted 1∶200). Slides were mounted using the ProLong Gold Antifade reagent with DAPI (Invitrogen). For paraffin-fixed tissues, 6 µM sections were prepared and after deparaffinization, antigen retrieval was carried out using a standard citrate protocol followed by permeabilization, staining with anti-human and anti-dengue antibodies, and mounted as described above. Slides were observed with a Nikon Eclipse TE2000-U microscope, and images were acquired and processed using a Nikon DS-Ri1 camera and the NIS-Elements Microscope Imaging Software (Nikon), respectively.

### Statistical analyses

For comparison of erythema index, temperature, and platelet measurements in infected and control humanized mice groups, a *t* test analysis was performed using GraphPad Prism v5.0.

## Results

### Infection and clinical signs in humanized mice

**Figure 1:**
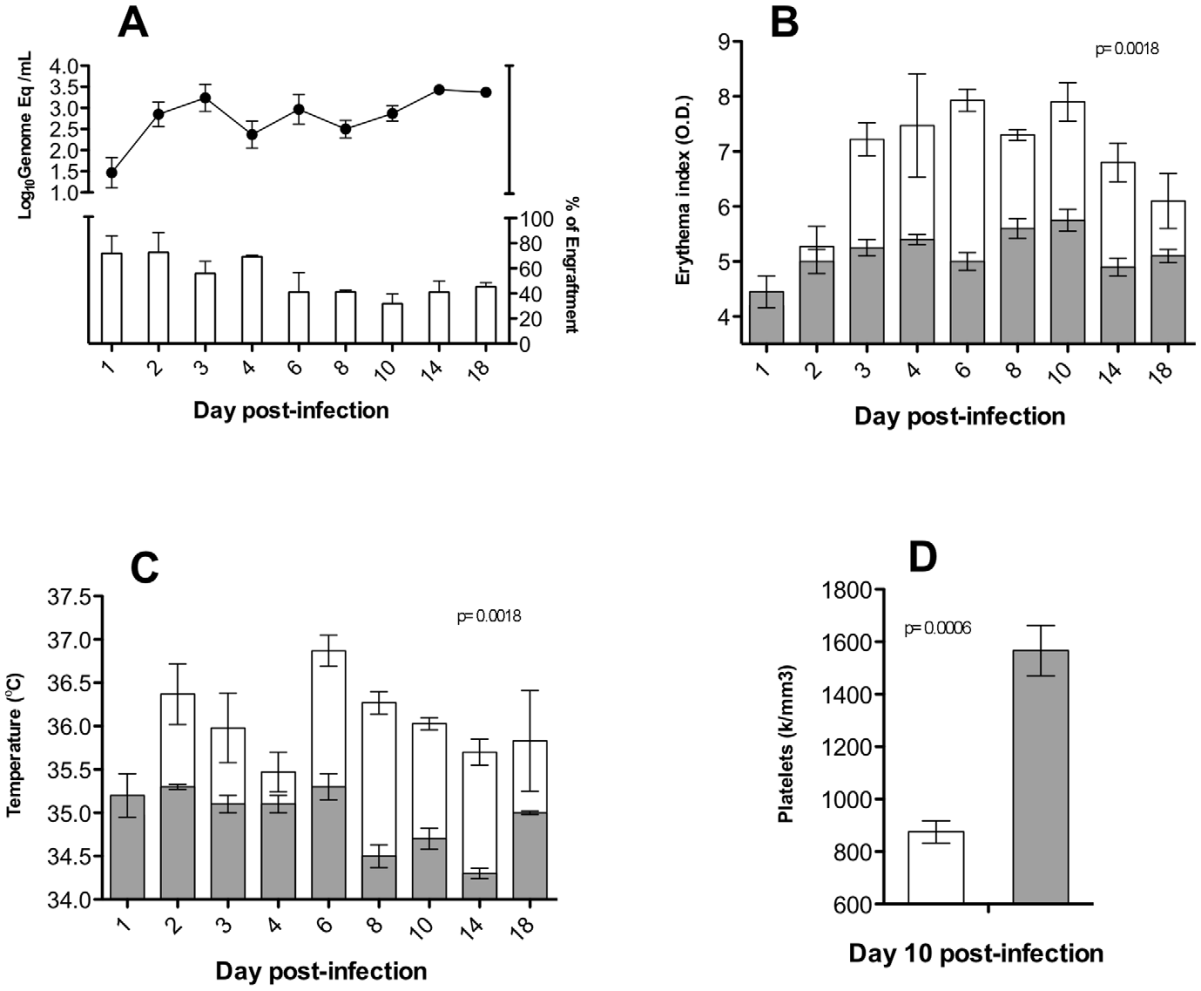
Humanized mice develop clinical signs as in humans. Groups of mice (n = 3) were intradermally inoculated with 9 log_10_ genome equivalents of the SE Asian strain K0049 of DEN-2, or with media only for control group, and viremia was measured in sera, at the indicated days post infection (p.i.) (**A**, upper panel); each point indicates the mean value of 3 mice and the standard error of the mean (SEM) is indicated by bars above and below each point. Engraftment levels of experimental groups at each time point are also shown (**A**, lower panel). Mock-inoculated control groups were always negative. Clinical signs were measured at the same time points, and the mean values of erythema (**B**) and fever (**C**) are shown. For thrombocytopenia, platelets were measured at day 10 p.i. (**D**). The statistical significance of the comparisons between infected (white boxes) and control groups (grey boxes) are shown in the upper portion of each panel.

Groups of humanized mice with engraftment levels ranging from 17 to 89% (percent of human CD45+ cells) were used in infection experiments as described previously [11]. We used the low passage DEN-2 strain K0049 (Southeast Asian genotype) for these infections, as we previously demonstrated that this strain has the most virulent phenotype [11]. Infected mice were followed for a period of up to 18 days post infection (p.i.) and clinical signs of infection were recorded at variable intervals (days 1, 2, 3, 4, 6, 8, 10, 14, and 18) before mice were euthanized. Our previous studies showed that dengue virus RNA was no longer detected on days 20–22 p.i. [11], so we decided to sacrifice mice at the latest on day 18 p.i., in order to detect infected cells before the viremia ended. Viremia was measured in sera of infected mice and the results showed a two-peak distribution as we observed previously [11], with one peak on day 3 p.i. and the second and highest around day 18 (**Fig. 1A**, upper panel). Human cell engraftment levels do not seem to influence the replication of this virus, since high viremia was observed even in experimental groups with low engraftment levels (day 10 p.i. **Fig. 1A**, lower panel). Because the mean of 3 mice is plotted for each day, only on day 1 p.i. the mean value dropped below the limit of detection. Mock-inoculated controls (media only) were always negative in the qRT-PCR. Infected mice also showed an increase in erythema index by day 3 p.i., peaking at days 6–8 and decreasing by day 18 (**Fig. 1B**) in accordance with our previous observations. In the case of fever, infected mice showed an increase in body temperature on the first day of infection, with small variations of about one degree Celsius along the time course (**Fig. 1C**), similar to what we previously observed [11]. In addition, we noted a significant reduction in the number of platelets in the infected mice at day 10 p.i. (**Fig. 1D**), an optimal time point for this measurement, based on our previous studies [11]. All clinical signs observed in the infected, humanized mice were statistically significant when compared with the mock-inoculated control groups.

### Infection with dengue occurs in human cells, in different mouse tissues

To study dengue virus tropism in the humanized mouse model, we analyzed the presence of dengue-infected cells in different tissues, at different days post infection. We collected blood, liver, lymph nodes, spleen and bone marrow from infected and control mice to identify the human cells that become infected and support dengue virus replication. We attempted to analyze replication in the liver, but no infected cells were found on any of the days tested (data not shown); we suspect that this is due to the lack of human hepatocytes in these mice. Since lymph nodes are absent or defective in the NSG mice, the tissues that were dissected in this area showed no consistent results and were not included here.

**Figure 2:**
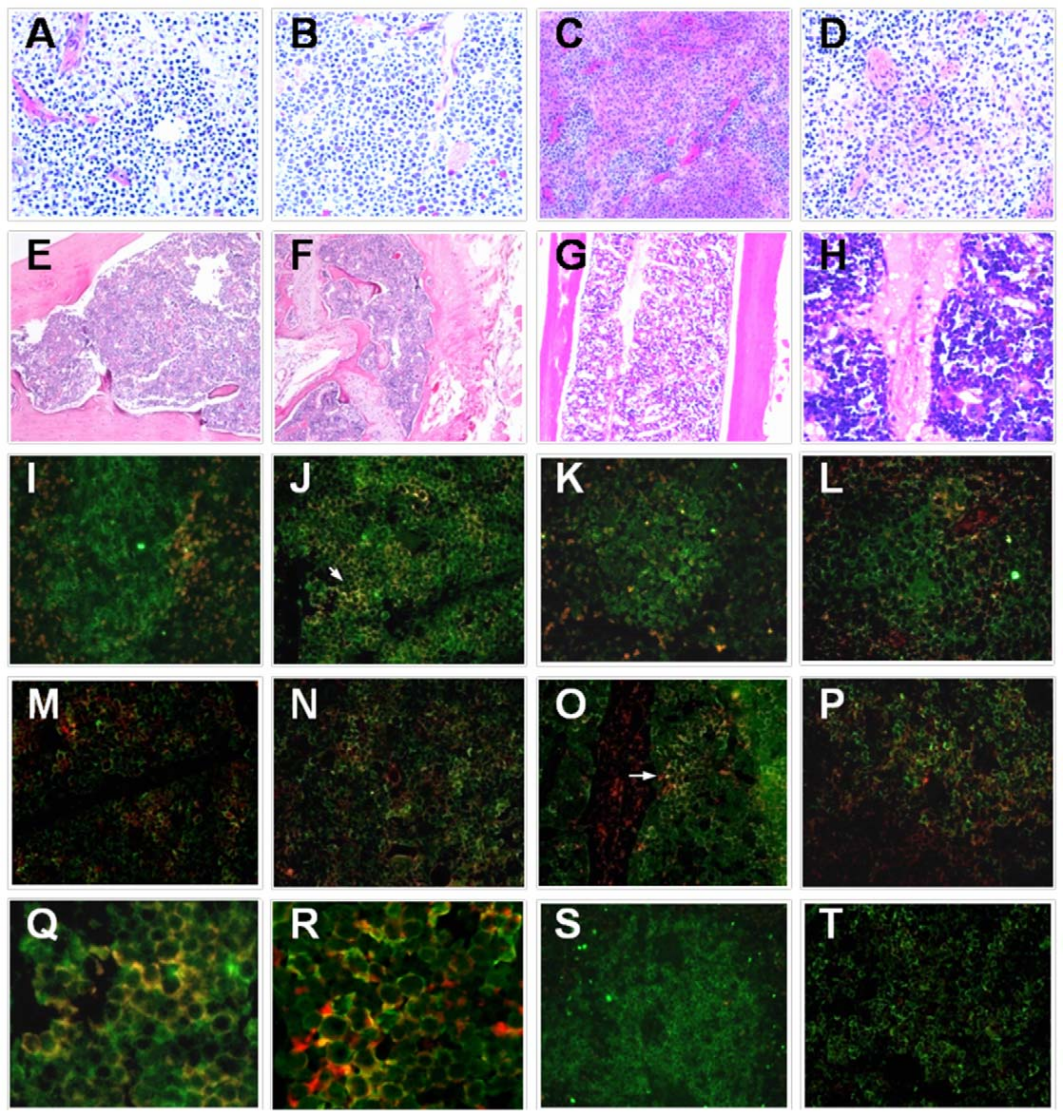
Dengue virus infects human cells in tissues of humanized mice. Morphology of spleen (SP) and bone marrow (BM) of humanized mice infected with dengue virus were analyzed by H&E staining (**A–H**). Tissue sections of SP and BM control mice (inoculated with media only) are shown in **A** and **B**, respectively (40× magnification), while for infected mice, SP sections are shown in **C** and **D** and different aspects of BM are shown in **E–H**; **C** and **E–G** are at 10×, **D** and **H** are at 40×. Infected human cells were analyzed by double staining with anti-human CD45 (green) and anti-DEN-2 3H5 Mab (red). Merged images (40×) of representative sections of SP and BM are shown in **I** and **M**, respectively (day 4 p.i.), **J** and **N** (day 10 p.i.), **K** and **O** (day 14 p.i.), and **L** and **P** (day 18 p.i.). Sections indicated by arrows in **J** and **O** are amplified in **Q** and **R**, respectively. Negative controls are shown in **S** (SP) and **T** (BM). Results presented here are representative images of 9 independent experiments (n = 3) performed at each time point.

We analyzed infected cells by means of immunofluorescence (IF) and flow cytometry assays. In the IF experiments, we first confirmed tissue architecture in the spleen and bone marrow by staining the tissue sections (5 µM) with hematoxylin and eosin; results showed a normal cell distribution and architecture in both tissues (**Fig. 2A–H**). Tissue sections were double stained for human CD45 positive cells (green) and for dengue virus serotype 2 (red), as described in Materials and Methods. For the spleen (**Fig. 2I–L**), we observed that during the first days of infection (**Fig. 2I**), double stained cells are dispersed outside the follicle-like structures (FLSs), while by day 10 p.i., intense double stained cells (orange-yellow color) are found inside the FLSs (**Fig. 2J and 2P**), which are mainly formed by B and T lymphocytes [7]. Later, at days 14 and 18 p.i, less intense staining double positive cells were observed, and most of the dengue positive cells appeared to be outside the FLSs. Similarly, for bone marrow (**Fig. 2M–P**) we observed positive cells for dengue antigen in leukocyte-rich areas, with more intense staining in cells located close to the central vein (**Fig. 2O and 2R**). Double positive cells increased in number by day 14 p.i and were still detectable by day 18. Stained sections from control mice showed only CD45+ cells and low background with the anti-E protein dengue antibody (**Fig. 2S–T**).

**Figure 3:**
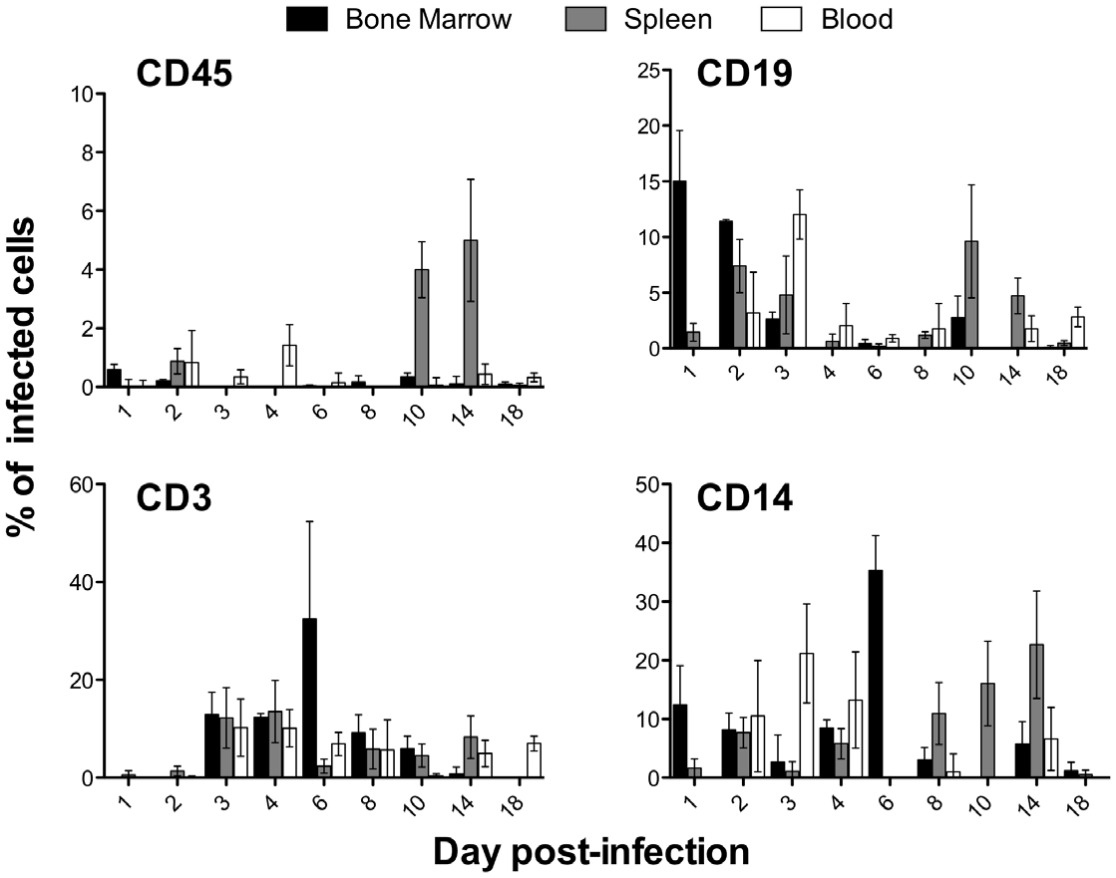
Human lymphocytes in mice are infected with dengue virus. Cell homogenates obtained at different time points p.i. from bone marrow (BM), spleen (SP), and blood (BL) of dengue-infected mice, were analyzed by flow cytometry to identify infected human cells. Cells were stained against human CD45 (general leukocyte marker), CD19 (B lymphocytes), CD3 (T lymphocytes), CD14 (monocytes/macrophages), and dengue prM protein. The percent of CD45^+^, CD45^+^-CD19^+^, CD45^+^-CD3^+^, and CD45^+^-CD14^+^ infected cells are shown. Values obtained from mock-inoculated (media only) mouse controls were subtracted from mean values of infected mice; plots are the mean of 3 mice (n = 3) at each time point, the SEM is indicated as bars.

For a finer identification of human cells infected by dengue virus in these mice, we performed flow cytometry analysis of the same tissues used in the IF analysis, but using antibodies against human B cells (CD19), T cells (CD3), monocytes/macrophages (CD14) and the general leukocyte marker CD45, in addition to an anti-dengue antibody specific for the prM protein. Human cells were analyzed in the lymphocyte gate defined as the region of forward and side scatter with the highest number of human CD45+ cells (Figures S1, S2, S3, S4, S5, S6, S7, S8, S9; one example per time point). The percent of infected cells for each specific lymphocyte subpopulation was obtained by dividing the number of dengue positive cells by the total number of cells in the lymphocyte subpopulation gate, and then multiplied by 100. Plotted results are the mean of results from 3 mice in each of 9 experimental groups, with the standard deviation of the mean (SEM) indicated also. Background staining obtained in mock-inoculated control mice was subtracted from values for infected mice. Results showed that human leukocytes in the bone marrow, spleen and in the peripheral blood are infected with dengue virus (**Fig. 3**). Within this population we found double positive cells for dengue and B lymphocytes, dengue and T lymphocytes, and dengue and monocytes/macrophages. Dengue positive B cells were observed primarily in the first 3 days p.i. and decreased dramatically by day 4, while dengue positive T cells appeared on day 3 and decreased to undetectable levels by day 18 p.i., except in blood. Of note was that the dengue positive monocyte/macrophage subpopulation presented a double peak distribution, resembling the viremia curve (**Fig. 1A**), with the first peak more evident in blood and bone marrow at days 3 and 6 p.i., respectively, and a second peak on days 10 and 14 p.i. in spleen. Since monocytes and macrophages are the main targets for dengue virus in human infections, these results are especially relevant to validating this model of disease. We attempted to measure the subpopulation of human dendritic cells positive for dengue antigen, but, because of the low frequency of this subpopulation in the lymphocyte gate, accurate analysis was not possible (data not shown), and further experiments to enrich this cell population in this mouse model will be necessary.

Studies performed with samples from autopsies of dengue patients showed dengue antigen and RNA in different organs or tissues [15]–[18]. Jessie et al [17] showed that macrophages, reactive lymphoid B cells, and peripheral lymphocytes were the major cell targets. In the present study we found histological evidence of infected cells in the spleen, particularly in the FLSs; using flow cytometry analysis, we were able to demonstrate the presence of B cells in this tissue at early and late stages of infection (days 2–3, and 10 and 14, respectively), in agreement with the autopsy studies. In addition, our results showing infected human cells in the bone marrow support previous observations of infected cells in long-term marrow cultures [19], [20] and in the bone marrow of autopsy samples [17], [21], [22]. Marrow cell suppression has been postulated to be involved in the leukopenia and thrombocytopenia observed in dengue fever patients [23]–[25]. One proposed mechanism of leukopenia is the activation of cross-reactive T cells, resulting in the production of bone marrow-suppressive cytokines. We observed infected T cells in the bone marrow of our mice that could have infiltrated in response to the inflammatory process and/or the infection of local cells, thus supporting such a mechanism of disease.

### Human cytokine and chemokine production in infected, humanized mice

**Figure 4:**
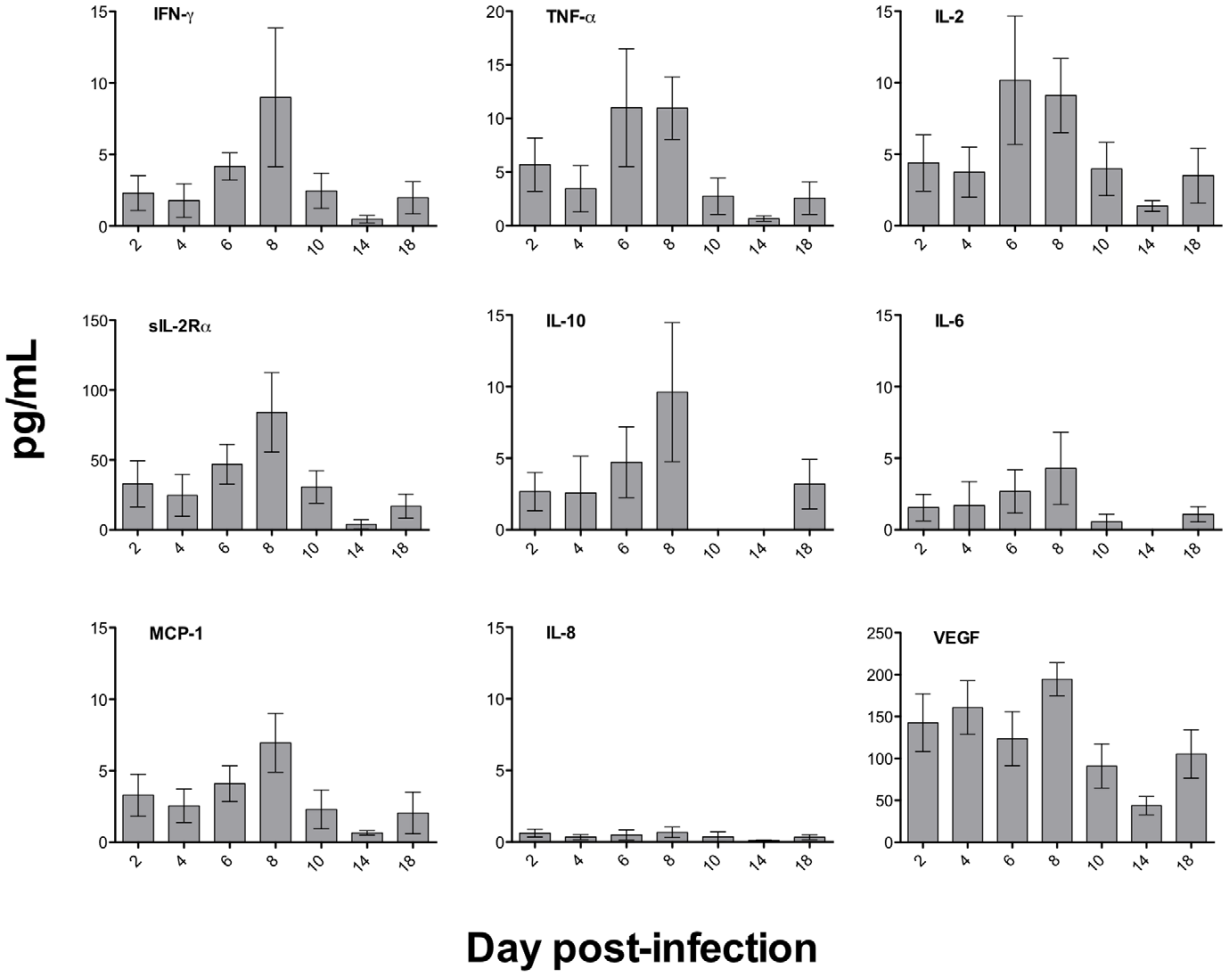
Humanized mice infected with dengue virus produce cytokines and chemokines. Human cytokines and chemokines (TNF-α, IFN-γ, IL-2, sIL-2R, IL-6, IL-8, IL-10, MCP-1 and VEGF) secreted by humans after dengue infection were measured in the sera of infected, humanized mice. Sera were obtained at different times p.i. and cytokine/chemokines measured in duplicate. Values for control sera, from mock-inoculated mice, were subtracted and plots indicate the mean of 3 mice (n = 3) at each time point; results were expressed in picograms per mL, with the SEM indicated as bars.

To evaluate the capacity of the human cells to produce cytokines and chemokines in response to dengue virus infection in these mice, and to compare this to the human response, we measured the levels of human TNF-α, IFN-γ, IL-2, sIL-2R, IL-6, IL-8, IL-10, MCP-1 and VEGF in the sera of infected and control mice at different time points. The fluorescently-labeled bead technology, in the multiplex format used here, allowed us to detect human cytokines and chemokines in very small volumes of sera obtained from infected mice. Results are presented as the mean value from 3 mice, after subtraction of background, which was the mean of the values in controls. Results showed that all of the measured cytokines or chemokines were secreted by human cells during infection, and that values for all peaked on day 8 p.i. (**Fig. 4**).

High levels of IFN-γ have been associated with severity in dengue-infected patients [26], [27]. This Th1 cytokine is produced by T cells and induces the production of inflammatory cytokines, and can activate mononuclear phagocytes (monocytes and dendritic cells), which could in turn produce other molecules such as TNF-α and platelet-activating factor, other mediators implicated in dengue pathogenesis [26], [28]. In the humanized mice we found high levels of IFN-γ by day 8 p.i., that could be related to the increased levels of TNF-α on days 6 and 8 p.i. Another Th1 cytokine is IL-2, a stimulator of cell grow and differentiation, which targets T and B cells, NK cells, macrophages and oligodendrocytes; it has been observed that levels of IL-2 along with other Th1 cytokines are elevated in dengue fever patients [29]. In humanized mice we found that IL-2 peaked at day 6 p.i., decreasing by day 14, and with a slight increase again by day 18. The soluble Il-2 receptor (sIL-2R) is also considered a marker of cell activation, and because it binds to soluble IL-2 it could regulate its action; however, in patients infected with dengue virus, it has been reported that sIL-2R levels are increased in serum or plasma [29], [30]. In dengue-infected mice we found increased levels of sIL-2R that peaked on day 8 p.i., similar to the curve observed for IL-2.

In addition, Th2 cytokines such as IL-10 have been associated with increased severity in dengue patients. IL-10 is an anti-inflammatory cytokine produced by monocytes and macrophages and it down-regulates the production of Th1 cytokines. The IL-10 profile in dengue patients showed low levels in dengue fever cases, while increased levels were observed in DHF patients [31]. In our model we observed a peak in IL-10 production by day 8 p.i., but it was undetectable on days 10–14 and increased again on day 18. In contrast, IL-6 was observed in low concentrations throughout: it appears on day 2, slightly increases on day 8, and returns to initial levels on day 18. This cytokine can act as a pro- and anti-inflammatory factor, and in dengue patients, increased levels have been observed in both DF and DHF cases [27], [31]. Two chemokines have been associated with increased severity in dengue cases: MCP-1 and IL-8 are increased in patients with DHF [27], [32]. MCP-1, along with TNF-α, has been associated with thrombocytopenia; in our mouse model we observed a significant decrease in the platelet count at day 10 p.i, which is proceeded by increased production of both TNF-α and MCP-1 on days 6 and 8 (Fig. 1D and 4, respectively). On the other hand, IL-8 levels in the serum of infected mice were always low, and we have no explanation for this; other studies have shown that IL-8 is low in DF patients, while it is consistently higher in DHF cases [27]. Finally, vascular endothelial growth factor (VEGF) seems to play an important role in the pathogenesis of dengue since it has been associated with vascular permeability in DHF patients [33], [34]; in the humanized mice we found that VEGF is elevated during the entire study period, peaking on day 8.

## Discussion

After several decades of research, there are important gaps in our understanding of dengue virus infection in humans and these gaps have slowed down the development of an effective treatment for dengue disease. A relevant, cost-effective, and predictable animal model is necessary to conduct fine and time-detailed studies in order to decipher the complex immunological mechanisms involved in dengue fever pathology. Non-human primates have been used in an attempt to accomplish this goal, but, outside of economical and ethical concerns, the main limitation of these models are in their biological relevance, because of low and transient viremias and the lack of consistent clinical signs or symptoms (as observed in human patients) [2]. Rodents are a more amenable small animal model of human diseases, and humanized mice have emerged as a powerful tool to study infectious disease, including dengue fever [6]. We previously demonstrated that mice transplanted with human hematopoietic progenitor cells can support dengue virus replication and develop clinical signs as occur in human patients [10], [11]. Here we demonstrated that dengue virus replicates in several tissues in the humanized NOD-*scid IL2Rγ* ^null^ mice, where human cells are located after transplantation. These results are in agreement with previous reports where dengue positive cells were found in the lymph nodes and spleen of human DHF cases [18]. We found that in addition to monocytes/macrophages, B and T leukocytes become infected and these infected cells could be detected as early as 24 hrs p.i. in the spleen, but more evidently in the bone marrow, after several days. B lymphocytes have been postulated as a target cell during virus infection, since dengue virus was recovered from B cells of PBMCs obtained from dengue patients generally [35] and from DHF patients specifically [36]; more recent flow cytometry analysis has shown some evidence for B cell infection, albeit by elimination of other cell subpopulations from pediatric patient samples [37]. Also, ex vivo-infected B cells, obtained from human PBMCs, support dengue virus replication as evidenced by negative-strand RNA amplification and structural and non-structural viral antigen detection [38]. Infected B cells were able to produce IL-6 and TNF-α, and viral replication and cytokine production were similar in monocytes from the same PBMCs. In our dengue fever model we observed that both cytokines are produced in infected mice, and it seems that B cells could play a role in supporting virus replication in the early stages of the infection (days 1–2 p.i.), especially in the bone marrow. Infected monocytes/macrophages are present in lower quantities early in infection, and are detected in the blood later, possibly acting as virus-transporting cells to different tissues; this could also explain the increasing levels of IL-6 and TNF-α in the sera of infected mice, after day 4 p.i. These results are relevant to natural human infection since both B cells and monocytes/macrophages express Fc receptors on their surface [39] and consequently they are potential targets for antibody-dependent enhancement of infection [40].

We found that human T cells in the humanized mice also are permissible for dengue virus infection. Contradictory reports have been published showing that T cells could either be or not infected with dengue virus [41]–[44]. We observed that infected T cells could be detected in all tissues analyzed by day 3 p.i. and this correlates with one of these studies. Mentor et al. [43] performed infections in human T cell lines and reported that dengue-positives cells could be detected by immunofluorescence staining at 20–60 hrs p.i., and virus could be detected in the cell culture supernatants by 40 hrs p.i.

We also analyzed liver samples of infected mice and were not able to detect infected cells although it has been widely reported that Kupffer cells in human liver could be target cells for dengue virus replication [16], [17], [45]. The reason we failed to detect dengue positive cells in the liver could be related to very low amounts of human Kupffer cells in humanized mouse liver, and further studies would be necessary to evaluate this possibility. Another organ in which we did not detect dengue virus previously is brain, and that is why we did not test that here [10]. Finally, we were not able to detect antibodies in infected mice. We attempted to measure total immunoglobulins (IgA+IgM+IgG) against dengue virus by ELISA as previously described [11], at days 10, 14 and 18 p.i, but results were negative in all cases. As we described previously, humanized mice transplanted with human umbilical cord blood have been characterized by the low or null production of antibodies, in part because of the lack of proper cytokine stimulation for B cells to survive in the murine environment [46], [47]. In our studies, we could speculate that in addition to the lack of the right microenviroment to expand and survive, B cells could be eliminated by other immune cells such as macrophages or cytotoxic T cells, since many of them are infected with dengue virus; however further experiments are needed to address this question.

In general we found that human lymphocytes are targets for dengue replication, and this is in agreement with several reports. Our results showed that dengue-infected CD45+ cells could be detected early during infection and among these, human lymphocytes, monocytes/macrophages and B cells seem to play a major role in supporting virus replication and dispersion. Other rodent models have been used to study DF or DHF (reviewed in [2], [48]), but these models require the use of biologically irrelevant factors, such as inoculation with very high virus doses of lab-adapted strains and using artificial inoculation routes that impede reaching robust and consistent conclusions about the complex mechanisms involved in dengue pathogenesis in humans. Humanized mouse models have thus emerged as a valuable tool to study dengue fever, using a more systematic and biologically appropriate approach [9], [10], [11], [49]; however, our model is the only one to develop clinical signs as in humans. In contrast with Kuruvilla et al [9], and Jaiswal et al [49], we use only low passage/non-adapted viruses and we have developed a systematic method to measure dengue clinical signs. In this study we demonstrated the presence of dengue antigen *in situ* by double immunostaining (in addition to the measurements of viremia in blood and clinical sings), in order to correlate the infection with a specific cell type, to overcome the limitations of interpretation based on cell morphology alone, as done by Balsitis et al. [18], or by quantification only of virus particles in tissues such as liver, as reported by Jaiswal et al [49], which could have been contaminated by infected cells from the blood. The methodology we developed to produce the humanized mice consistently produces high engraftment levels and these mice support dengue virus replication, develop signs of disease, and infected human cells produce cytokines and chemokines as observed in humans. This provides evidence for an innate immune response to dengue virus infection in these mice. We are currently attempting to improve the adaptive immune response by adding human cytokines to these mice, to maintain B and T cell function throughout the infection process, and to use a more natural route and dose of infection, to get transmission via mosquito bite. This should allow us to study in finer detail the complex mechanisms that produce dengue fever, especially after natural transmission by mosquito bite.

Histopathological studies of dengue patients have been historically difficult because of many factors, including the fact that tissue samples are difficult to obtain from patients with dengue fever only, and that fatal cases are rare and may occur in remote areas, and it is often difficult to obtain autopsy samples [50]. In addition, results obtained from samples that were collected, processed and analyzed in diverse forms, make the interpretation of pathological findings difficult or inconsistent. The humanized model presented here represents an efficient way to analyze in a timely fashion the tropism of dengue virus and the role of specific cell populations in the development of dengue fever. In summary, and without ignoring that the humanized mouse is still a model with limitations, results shown here demonstrate that the humanized NOD-*scid IL2Rγ* ^null^ mice developed in our laboratory can be amenable to studying dengue virus infection and pathogenesis.

Note added in proof: A recent study by Baclig et al., (Southeast Asian J. Trop. Med. Public Health, 2010, 41(6):1352–1358) confirmed that many, if not most, of dengue-infected peripheral blood cells from patients are CD19+ or B cells.

## Supporting Information

Figure S1 **Representative flow cytometry analysis of dengue infected human cells in the bone marrow of a humanized mouse (day 1 p.i.).** Tissue was collected and processed as described in materials and methods at the indicated time point. Cells were stained for human B lymphocytes (CD19), T lymphocytes (CD3), monocytes/macrophages (CD14), human leukocytes (CD45), and for dengue virus (E protein). Double positive cells for each cell subpopulation in the CD45 gate from each mouse (n = 3) were determined and the mean value was plotted in Figure 3 (see text for details).
(TIF)

Figure S2 **Representative flow cytometry analysis of dengue infected human cells in the bone marrow of a humanized mouse (day 2 p.i.).** Tissue was collected and processed as described in materials and methods at the indicated time point. Cells were stained for human B lymphocytes (CD19), T lymphocytes (CD3), monocytes/macrophages (CD14), human leukocytes (CD45), and for dengue virus (E protein). Double positive cells for each cell subpopulation in the CD45 gate from each mouse (n = 3) were determined and the mean value was plotted in Figure 3 (see text for details).
(TIF)

Figure S3 **Representative flow cytometry analysis of dengue infected human cells in the bone marrow of a humanized mouse (day 3 p.i.).** Tissue was collected and processed as described in materials and methods at the indicated time point. Cells were stained for human B lymphocytes (CD19), T lymphocytes (CD3), monocytes/macrophages (CD14), human leukocytes (CD45), and for dengue virus (E protein). Double positive cells for each cell subpopulation in the CD45 gate from each mouse (n = 3) were determined and the mean value was plotted in Figure 3 (see text for details).
(TIF)

Figure S4 **Representative flow cytometry analysis of dengue infected human cells in the bone marrow of a humanized mouse (day 4 p.i.).** Tissue was collected and processed as described in materials and methods at the indicated time point. Cells were stained for human B lymphocytes (CD19), T lymphocytes (CD3), monocytes/macrophages (CD14), human leukocytes (CD45), and for dengue virus (E protein). Double positive cells for each cell subpopulation in the CD45 gate from each mouse (n = 3) were determined and the mean value was plotted in Figure 3 (see text for details).
(TIF)

Figure S5 **Representative flow cytometry analysis of dengue infected human cells in the bone marrow of a humanized mouse (day 6 p.i.).** Tissue was collected and processed as described in materials and methods at the indicated time point. Cells were stained for human B lymphocytes (CD19), T lymphocytes (CD3), monocytes/macrophages (CD14), human leukocytes (CD45), and for dengue virus (E protein). Double positive cells for each cell subpopulation in the CD45 gate from each mouse (n = 3) were determined and the mean value was plotted in Figure 3 (see text for details).
(TIF)

Figure S6 **Representative flow cytometry analysis of dengue infected human cells in the bone marrow of a humanized mouse (day 8 p.i.).** Tissue was collected and processed as described in materials and methods at the indicated time point. Cells were stained for human B lymphocytes (CD19), T lymphocytes (CD3), monocytes/macrophages (CD14), human leukocytes (CD45), and for dengue virus (E protein). Double positive cells for each cell subpopulation in the CD45 gate from each mouse (n = 3) were determined and the mean value was plotted in Figure 3 (see text for details).
(TIF)

Figure S7 **Representative flow cytometry analysis of dengue infected human cells in the bone marrow of a humanized mouse (day 10 p.i.).** Tissue was collected and processed as described in materials and methods at the indicated time point. Cells were stained for human B lymphocytes (CD19), T lymphocytes (CD3), monocytes/macrophages (CD14), human leukocytes (CD45), and for dengue virus (E protein). Double positive cells for each cell subpopulation in the CD45 gate from each mouse (n = 3) were determined and the mean value was plotted in Figure 3 (see text for details).
(TIF)

Figure S8 **Representative flow cytometry analysis of dengue infected human cells in the bone marrow of a humanized mouse (day 14 p.i.).** Tissue was collected and processed as described in materials and methods at the indicated time point. Cells were stained for human B lymphocytes (CD19), T lymphocytes (CD3), monocytes/macrophages (CD14), human leukocytes (CD45), and for dengue virus (E protein). Double positive cells for each cell subpopulation in the CD45 gate from each mouse (n = 3) were determined and the mean value was plotted in Figure 3 (see text for details).
(TIF)

Figure S9 **Representative flow cytometry analysis of dengue infected human cells in the bone marrow of a humanized mouse (day 18 p.i.).** Tissue was collected and processed as described in materials and methods at the indicated time point. Cells were stained for human B lymphocytes (CD19), T lymphocytes (CD3), monocytes/macrophages (CD14), human leukocytes (CD45), and for dengue virus (E protein). Double positive cells for each cell subpopulation in the CD45 gate from each mouse (n = 3) were determined and the mean value was plotted in Figure 3 (see text for details).
(TIF)
