# Supplementary figures and images for: Dengue Virus Tropism in Humanized Mice Recapitulates Human Dengue Fever
Source: PLoS One. 2011 Jun 10;6(6):e20762. doi: 10.1371/journal.pone.0020762 (PMC3112147; doi:10.1371/journal.pone.0020762)

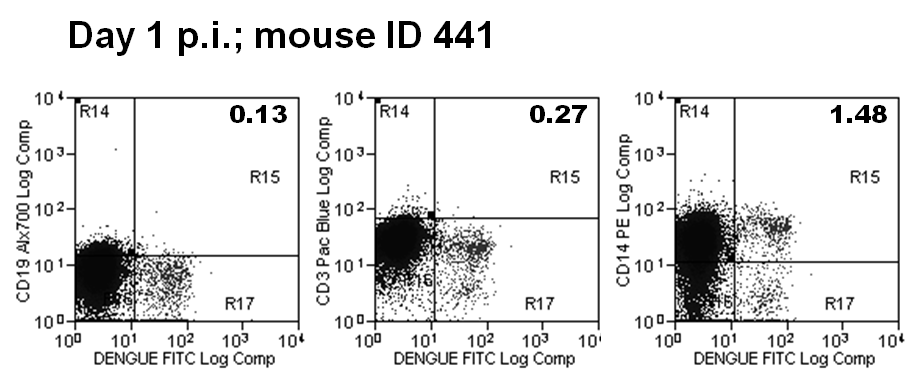

Supplement: Figure S1 — Representative flow cytometry analysis of dengue infected human cells in the bone marrow of a humanized mouse (day 1 p.i.). Tissue was collected and processed as described in materials and methods at the indicated time point. Cells were stained for human B lymphocytes (CD19), T lymphocytes (CD3), monocytes/macrophages (CD14), human leukocytes (CD45), and for dengue virus (E protein). Double positive cells for each cell subpopulation in the CD45 gate from each mouse (n = 3) were determined and the mean value was plotted in Figure 3 (see text for details). (TIF) [file pone.0020762.s001.tif]

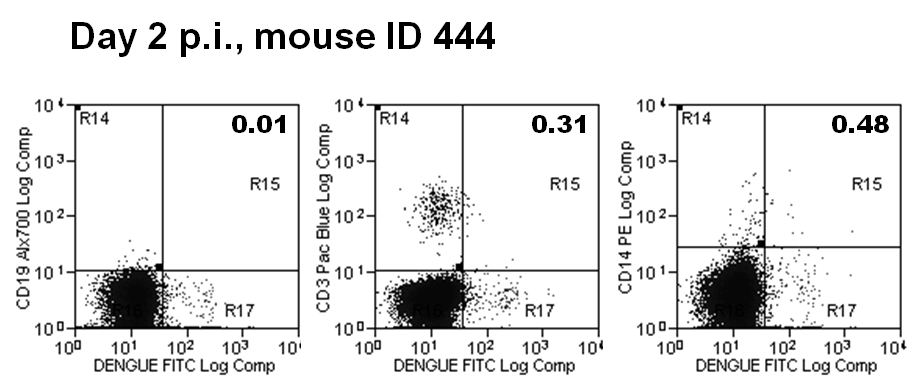

Supplement: Figure S2 — Representative flow cytometry analysis of dengue infected human cells in the bone marrow of a humanized mouse (day 2 p.i.). Tissue was collected and processed as described in materials and methods at the indicated time point. Cells were stained for human B lymphocytes (CD19), T lymphocytes (CD3), monocytes/macrophages (CD14), human leukocytes (CD45), and for dengue virus (E protein). Double positive cells for each cell subpopulation in the CD45 gate from each mouse (n = 3) were determined and the mean value was plotted in Figure 3 (see text for details). (TIF) [file pone.0020762.s002.tif]

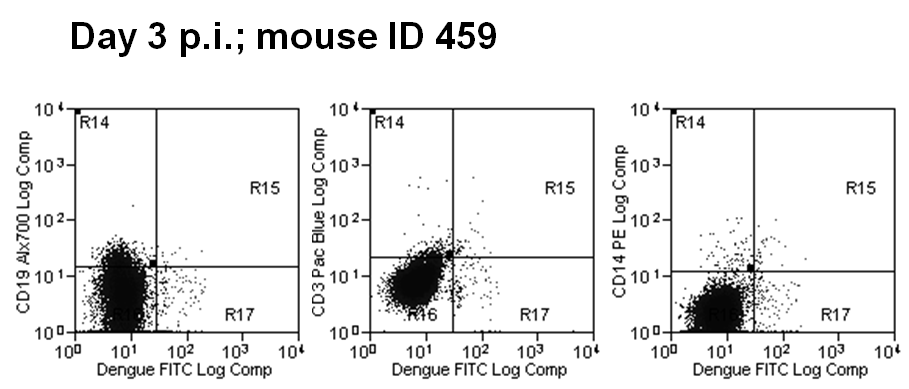

Supplement: Figure S3 — Representative flow cytometry analysis of dengue infected human cells in the bone marrow of a humanized mouse (day 3 p.i.). Tissue was collected and processed as described in materials and methods at the indicated time point. Cells were stained for human B lymphocytes (CD19), T lymphocytes (CD3), monocytes/macrophages (CD14), human leukocytes (CD45), and for dengue virus (E protein). Double positive cells for each cell subpopulation in the CD45 gate from each mouse (n = 3) were determined and the mean value was plotted in Figure 3 (see text for details). (TIF) [file pone.0020762.s003.tif]

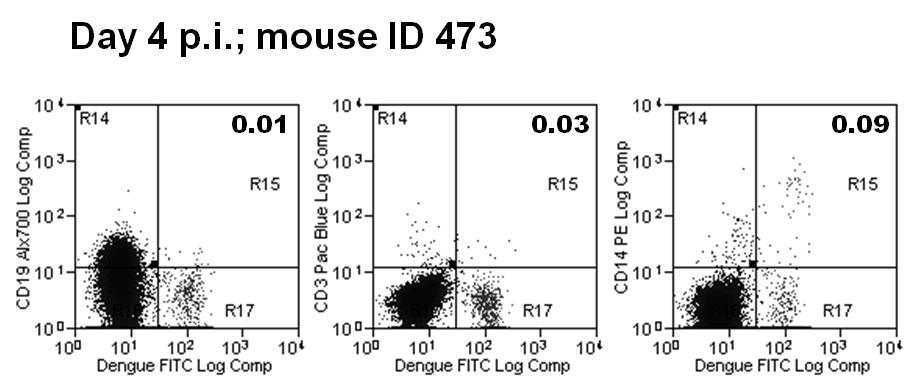

Supplement: Figure S4 — Representative flow cytometry analysis of dengue infected human cells in the bone marrow of a humanized mouse (day 4 p.i.). Tissue was collected and processed as described in materials and methods at the indicated time point. Cells were stained for human B lymphocytes (CD19), T lymphocytes (CD3), monocytes/macrophages (CD14), human leukocytes (CD45), and for dengue virus (E protein). Double positive cells for each cell subpopulation in the CD45 gate from each mouse (n = 3) were determined and the mean value was plotted in Figure 3 (see text for details). (TIF) [file pone.0020762.s004.tif]

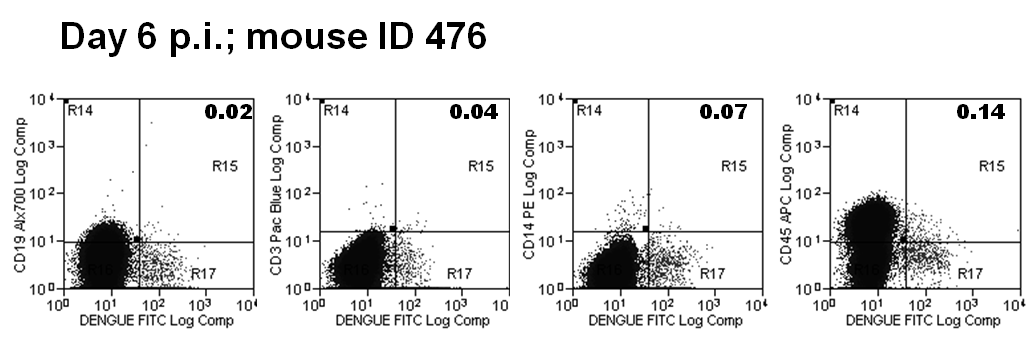

Supplement: Figure S5 — Representative flow cytometry analysis of dengue infected human cells in the bone marrow of a humanized mouse (day 6 p.i.). Tissue was collected and processed as described in materials and methods at the indicated time point. Cells were stained for human B lymphocytes (CD19), T lymphocytes (CD3), monocytes/macrophages (CD14), human leukocytes (CD45), and for dengue virus (E protein). Double positive cells for each cell subpopulation in the CD45 gate from each mouse (n = 3) were determined and the mean value was plotted in Figure 3 (see text for details). (TIF) [file pone.0020762.s005.tif]

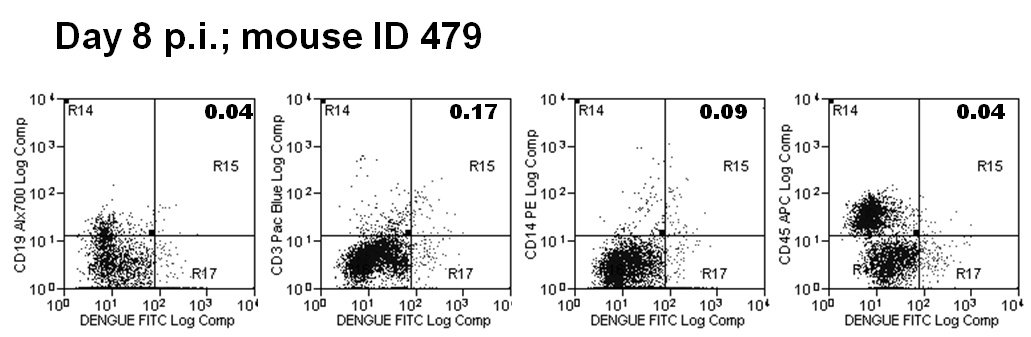

Supplement: Figure S6 — Representative flow cytometry analysis of dengue infected human cells in the bone marrow of a humanized mouse (day 8 p.i.). Tissue was collected and processed as described in materials and methods at the indicated time point. Cells were stained for human B lymphocytes (CD19), T lymphocytes (CD3), monocytes/macrophages (CD14), human leukocytes (CD45), and for dengue virus (E protein). Double positive cells for each cell subpopulation in the CD45 gate from each mouse (n = 3) were determined and the mean value was plotted in Figure 3 (see text for details). (TIF) [file pone.0020762.s006.tif]

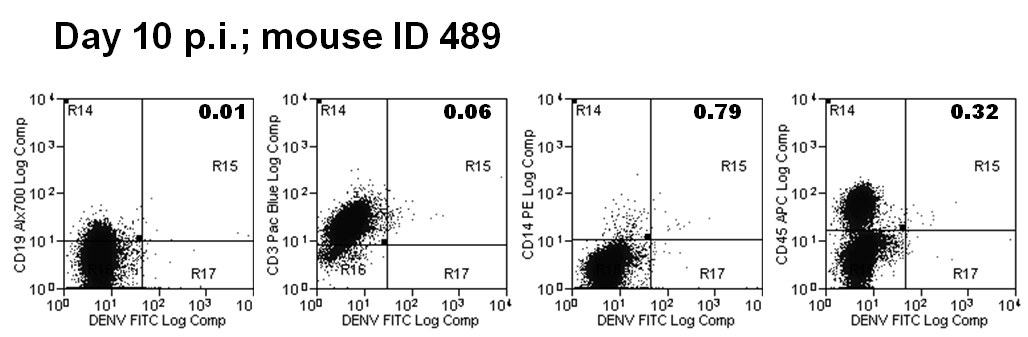

Supplement: Figure S7 — Representative flow cytometry analysis of dengue infected human cells in the bone marrow of a humanized mouse (day 10 p.i.). Tissue was collected and processed as described in materials and methods at the indicated time point. Cells were stained for human B lymphocytes (CD19), T lymphocytes (CD3), monocytes/macrophages (CD14), human leukocytes (CD45), and for dengue virus (E protein). Double positive cells for each cell subpopulation in the CD45 gate from each mouse (n = 3) were determined and the mean value was plotted in Figure 3 (see text for details). (TIF) [file pone.0020762.s007.tif]

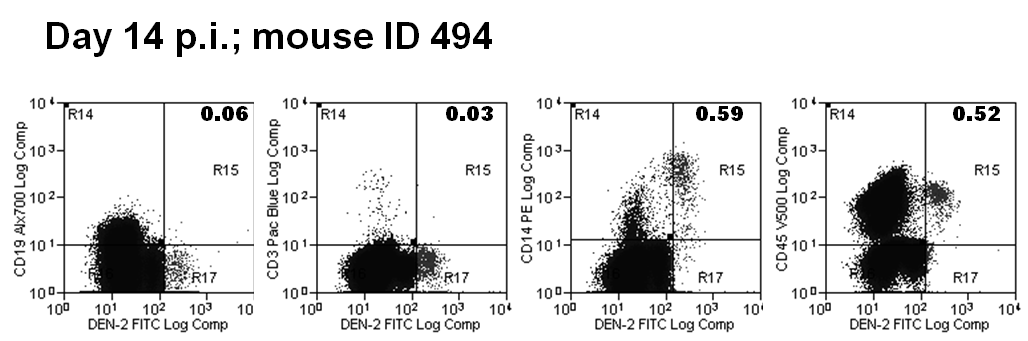

Supplement: Figure S8 — Representative flow cytometry analysis of dengue infected human cells in the bone marrow of a humanized mouse (day 14 p.i.). Tissue was collected and processed as described in materials and methods at the indicated time point. Cells were stained for human B lymphocytes (CD19), T lymphocytes (CD3), monocytes/macrophages (CD14), human leukocytes (CD45), and for dengue virus (E protein). Double positive cells for each cell subpopulation in the CD45 gate from each mouse (n = 3) were determined and the mean value was plotted in Figure 3 (see text for details). (TIF) [file pone.0020762.s008.tif]

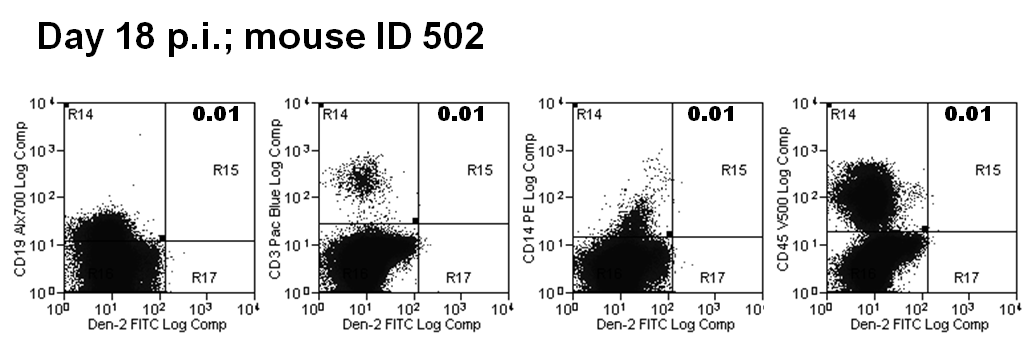

Supplement: Figure S9 — Representative flow cytometry analysis of dengue infected human cells in the bone marrow of a humanized mouse (day 18 p.i.). Tissue was collected and processed as described in materials and methods at the indicated time point. Cells were stained for human B lymphocytes (CD19), T lymphocytes (CD3), monocytes/macrophages (CD14), human leukocytes (CD45), and for dengue virus (E protein). Double positive cells for each cell subpopulation in the CD45 gate from each mouse (n = 3) were determined and the mean value was plotted in Figure 3 (see text for details). (TIF) [file pone.0020762.s009.tif]
